# Tranexamic acid adverse reactions: a brief summary for internists and emergency doctors

**DOI:** 10.1186/s12948-020-00131-8

**Published:** 2020-09-03

**Authors:** Giuseppe Murdaca, Monica Greco, Chiara Vassallo, Sebastiano Gangemi

**Affiliations:** 1 Clinical Immunology Unit, Department of Internal Medicine, University of Genoa and Ospedale Policlinico San Martino, Viale Benedetto XV, n. 6, 16132 Genoa, Italy; 2 School and Operative Unit of Allergy and Clinical Immunology, Department of Clinical and Experimental Medicine, University of Messina, 98125 Messina, Italy

**Keywords:** Tranexamic acid, Drug hypersensitivity, Anaphylaxis, Ethamsylate

## Abstract

Tranexamic acid (TXA) is a synthetic lysine analogue that is well known as antifibrinolytic agent. It can reduce blood loss in clinical use, especially in conditions where fibrinolysis or hyperfibrinolysis are involved, such as trauma or surgery. Moreover, TXA has been approved as second-line prophylactic therapy for hereditary angioedema and further data have been published about a possible use of TXA as maintenance treatment for nonhistaminergic angioedema and treatment for episodes of bradykinin-mediated angioedema induced by ACE inhibitors. TXA can be administered through several routes: orally, topically, or intravenously. Although, it is a drug with a very high safety profile, in few cases hypersensitivity reactions have been described occurring with different clinical manifestations. Ethamsylate can be an alternative in TXA sensitized patients. In this brief article we describe TXA adverse reactions and current protocols which have been proposed to help clinicians to diagnose TXA hypersensitivity.

## Key points

- Tranexamic acid (TXA) is a synthetic lysine analogue that is well known as antifibrinolytic agent. Moreover, TXA is approved as a second-line prophylactic therapy for hereditary angioedema.
- TXA is largely used in surgical and emergency settings and potential adverse reactions may be of interest not only for allergologist.
- TXA adverse reactions may vary from cutaneous to anaphylactic reactions.
- Ethamsylate can be an alternative in case of TXA adverse reactions.

## Background

### Mechanisms of action and clinical pharmacology

Tranexamic acid (TXA) is a synthetic lysine analogue that is well known as antifibrinolytic agent. Plasminogen and plasmin are the central enzyme in fibrinolysis. Plasminogen has a lysine binding sites that are essential for maintaining a closed conformation in circulation and binding to fibrin and cell surface receptors. Upon binding to the targets, plasminogen is activated to plasmin by the fibrin-bound tissue plasminogen activator or the cell receptor–bound urokinase plasminogen activator. TXA exerts its antifibrinolytic effect by reversibly blocking lysine binding sites on plasminogen thus preventing plasmin from interacting with lysine residues on the fibrin polymer and subsequent fibrin degradation [1]. This can explain the effect of reducing blood loss in clinical use, especially in conditions where fibrinolysis or hyperfibrinolysis is a factor, such as trauma or surgery [2]. TXA has showed protective effects on the endothelium and beneficial modulation of inflammation and other responses following ischaemia and reperfusion. These observed activities suggest that TXA might be involved in an inflammatory pathway indeed plasmin plays roles in inflammation, angiogenesis, and wound healing [3]. Moreover, TXA is also able to reduce bradykinin levels. That explain his efficacy when used in those clinical status caused by a dysregulation of this pathway [4]. TXA can be administered through several routes—orally, topically, or intravenously. The oral bioavailability of tranexamic acid is approximately 34% and the pharmacokinetics are unaffected by the presence of food in the gastrointestinal tract. Tranexamic acid is minimally bound to plasma proteins (≈3%) at therapeutic plasma concentrations (5–10 mg/L), and this appears to be fully accounted for by binding to plasminogen. TXA is cleared through the renal system, which means its dosage needs to be adjusted appropriately for patients who are suffering from chronic kidney disease [5].

### Therapeutic indications

At first, TXA has been approved for tooth extractions in patients with hemophilia, to reduce or prevent hemorrhage and reduce the need for replacement therapy. Afterwards, TXA has been widely used to reduce blood loss in surgery, first cardiac surgery and joint replacement [6, 7], and subsequently in many surgical fields (otorhinolaryngology, neurosurgery, gynecology, etc.). The benefic effects of TXA have been applied in patients with acute severe bleeding. The randomized controlled trial WOMAN proved that TXA reduces mortality from bleeding in patients with post-partum hemorrhage [8]. The CRASH-2 trial demonstrated that tranexamic acid significantly reduces death due to bleeding and all-cause mortality, with no increase in vascular occlusive events in trauma patients [9].In this field, TXA has shown to have an excellent safety profile and to be cost effective; furthermore the benefit of his administration was found when it is given within 3 h after injury [10]. The effective of early administration of TXA was also demonstrated in traumatic brain injury [11] and intracerebral hemorrhage [12]. Another helpful use of TXA has been with angio-eodema and urticaria. Indeed, in the past antifibrinolytic agents such as TXA or epsilon aminocaproic acid were used as first-line therapies for these patients. To date, although newer targeted therapies, such as intravenous plasma-derived C1 inhibitor (Cinryze) have been come out, TXA is still approved as a second-line prophylactic therapy for hereditary angiooedema [13] and further data have been published about a possible use of TXA as maintenance treatment for nonhistaminergic angioedema [14] and treatment for episodes of bradykinin-mediated angioedema induced by ACE inhibitors [15]. Other additional indications are: menorrhagia, epistaxis and hyphema.

### Tranexamic acid hypersensitivity

Fifteen year ago, the first case of anaphylaxis caused by TXA was reported [16]. Thereafter, the interest in TXA hypersensitivity arose [17]. To date several cases of TXA adverse reactions have been reported especially in perioperative medicine subsets. Symptoms of TXA hypersensitivity may range from mild as skin rash, itching and/or urticaria to severe including wheezing, hypotension and shock [18], thus reflecting the different pathogenetic mechanisms involved. Indeed, both IgE and cellular-mediated reactions have been hypothesized. Although, the majority of documented reactions are IgE-mediated type 1 hypersensitivity reactions, fixed drug eruption (FDE), which underlies a T-cell mediated mechanism, has also been described [19]. In their case, authors not only confirmed TXA as the causative agent, but also reported the failure to desensitize the patient, maybe to the complexity of pathogenetic mechanisms involved in FDE. To sum up, hypersensitivity reactions to TXA maybe IgE-mediated or cellular mediated and clinical symptoms and laboratory tests are crucial to address clinician suspects. For this purpose, Li et al. [20] suggested a protocol for the investigation of a suspected anaphylactic reaction to TXA. Firstly, an accurate and complete medical history should be collected, thus focusing on previous suspected drug exposures. In an anaphylactic reaction is suspected, tryptase levels 30 to 120 min and 24 h after the reaction should be collected. Cutaneous tests are then needed. Authors suggested to perform skin prick tests (SPT) 100 mg/mL and intradermal tests (IDT) with 0.01 mg/mL and 0.1 mg/mL. The diagnosis could then be confirmed with a graded provocation test. Regarding this aspect, the oral route is should be preferred because of its higher safety profile, noticing that TXA bioavailability is less than 40%. On the other hand, in case of an unlikely type-I hypersensitivity reaction, allergology work-up should include delayed IDTs or patch tests.

## Discussion

Major drugs involved in perioperative adverse reactions are antibiotics, neuromuscular blocking agents (NMBAs), hypnotic drugs, chlorhexidine, dyes and latex [21]. Although TXA is not considered to be part of the above-mentioned list, adverse reactions including anaphylaxis have been reported. To date standardized protocols have been proposed in order to help clinicians to avoid unexpected reactions especially in the emergency and perioperative settings. Focusing on this topic, although TXA is considered the first treatment option for hemorrhagic conditions, it could be not well tolerated by some patients. Therefore, alternatives to TXA are aminocaproic acid and etamsylate. The former is actually not recommended as it shares the lysine compound with TXA. On the other hand, ethamsylate demonstrated to be a good substitute. Ethamsylate is a synthetic hemostatic drug which improves platelet adhesiveness, through P-selectin-dependent mechanisms and restoring capillary resistance [22]. Moreover, it works on cyclooxygenase metabolism, thus decreasing thromboxane A2 levels and prostacyclin biosynthesis, resulting in anti-inflammatory action. To date, ethamsylate major indications are menorrhagia, ﻿periventricular hemorrhage (PVH) in very low birth weight babies, surgical postsurgical bleeding. Concerning ethamsylate safety profile, several studies demonstrated that although it may occasionally induce nausea and headache, no major adverse events are associated with its usage. However, concerns regarding a possible association with thrombotic events, such as deep vein thrombosis raised. To date several studies investigated on this subject [23–25], but no evidence of deep vein thrombosis was found in ethamsylate cohorts. To conclude, ethamsylate proved to be effective in several conditions and the risk of allergic reaction in TXA sensitized patients is insignificant, so it can be considered as a good substitute, even if TXA remains the first-line treating option. Other options different to ethamsylate depend on the specific hemorrhagic condition, thus including surgical approaches.

## Conclusions

TXA is a synthetic lysine analogue that is well known as antifibrinolytic agent. This drug can be used in several conditions, especially in bleeding disorders, thus explaining its significant role among acute and perioperative settings. TXA is a drug with a very high safety profile and in some Countries it is considered to be an over the counter product. Indeed, it’s use in acute and perioperative settings is still fundamental in clinical practice. However, in few cases hypersensitivity reactions have been described occurring with different clinical manifestations. In this brief article we highlight current protocols proposed to help clinicians to diagnose TXA hypersensitivity. Finally, although TXA remains the first line therapy in case of hemorrhagic conditions ethamsylate, another synthetic non-lysine molecule can be an alternative option in TXA sensitized patients.

## Data Availability

Not applicable.
